# 异基因造血干细胞移植治疗DEK-NUP214融合基因阳性急性髓系白血病16例临床观察

**DOI:** 10.3760/cma.j.issn.0253-2727.2023.12.014

**Published:** 2023-12

**Authors:** 晶 夏, 晔 赵, 小津 吴, 惠英 仇, 晓文 唐, 荧 王, 正明 金, 瞄 苗, 骁 马, 德沛 吴, 苏宁 陈, 峰 陈

**Affiliations:** 1 苏州大学附属第一医院血液科，江苏省血液研究所，国家血液系统疾病临床医学研究中心，苏州 215000 Department of Hematology, the First Affiliated Hospital of Soochow University, Jiangsu Institute of Hematology, National Clinical Research Center for Hematologic Diseases, Suzhou 215000, China; 2 苏州弘慈血液病医院血液科，苏州 215000 Department of Hematology, Soochow Hopes Hematology Hospital, Suzhou 215000, China

约60％的急性髓系白血病（AML）存在克隆性染色体结构、数量异常，并发生与细胞分化、增殖有关的基因突变或表达异常。t（6;9）（p23;q34）是AML中不常见的一类染色体异常，在成人AML中占比为1％～5％，在儿童AML中占比为1％～2％，也有慢性髓性白血病及骨髓增生异常综合征（MDS）的个案报道[1]–[2]。DEK-NUP214融合基因由6号染色体上的DEK基因与9号染色体上的NUP214基因产生基因重排形成。DEK-NUP214融合基因阳性AML患者复发率高、预后差，因此世界卫生组织（WHO）分型将其定义为一种独特的临床类型。以往研究结果表明尽早实施异基因造血干细胞移植（allo-HSCT）能够克服DEK-NUP214对AML患者的不良影响[3]–[4]。本研究中，我们对近年来在我院接受allo-HSCT治疗的16例DEK-NUP214融合基因阳性AML患者进行回顾性分析。

## 病例与方法

一、病例

本研究纳入2017年7月至2022年10月苏州大学附属第一医院和苏州弘慈血液病医院接受allo-HSCT的16例DEK-NUP214融合基因阳性AML患者。中位发病年龄为38（23～58）岁，女7例、男9例，全部病例均符合WHO（2016）的AML诊断标准。

二、融合基因及基因突变检测

取骨髓标本3 ml，分离单个核细胞。按照TRIzol方法提取总RNA，反转录获得cDNA，多重巢式PCR技术进行急性白血病有关的29类融合基因（包括DEK-NUP 214）检测。抽提基因组DNA，建立包含DNMT3A、CEBPA、KIT、FLT3、NRAS等51个白血病关联的高频基因IonAmpliSeq文库，采用ABI Ion Torrent S5测序仪进行检验。

三、染色体核型分析

骨髓细胞历经24～48 h培养、制片、R显带，分析中期细胞20个，遵从《人类细胞遗传学国际命名体制（ISCN）（2016）》进行核型分析。

四、诱导及巩固治疗方案

诱导化疗方案选择IA（去甲氧柔红霉素+阿糖胞苷）、地西他滨+HAAG（高三尖杉酯碱+阿克拉霉素+阿糖胞苷+G-CSF）或HA（高三尖杉酯碱+阿糖胞苷）联合Bcl-2抑制剂（维奈克拉），部分病例存在FLT3基因突变，加用索拉非尼或富马酸吉瑞替尼靶向治疗。巩固方案包括原诱导方案或中剂量阿糖胞苷方案（2 g/m^2^，每12 h 1次×3 d）等。血象恢复后行骨髓穿刺评估疾病缓解情况，如获得完全缓解（CR）则给予巩固治疗，未获得CR的患者再行诱导化疗。

五、移植方案

全部病例均采用改良BUCY预处理方案。采用钙调磷酸酶抑制剂+短程甲氨蝶呤预防移植物抗宿主病（GVHD）。无关供者全相合、单倍体移植患者在此基础上联合霉酚酸酯（MMF）、兔抗人胸腺细胞球蛋白（rATG）。其他移植有关并发症的预防及治疗参见文献[5]。

六、随访

随访截至2023年1月15日，中位随访时间为22.8（3.2～66.9）个月，无失访病例。采用查阅门诊/住院病历和电话联系获得患者移植后生存资料。总生存（OS）时间设定为造血干细胞回输到随访截止或死亡的时间，无病生存（DFS）时间定义为移植后获得CR至疾病复发、随访截止或死亡的时间。

七、统计学处理

使用SPSS16.0软件进行统计学分析，使用Kaplan-Meier生存曲线分析DFS和OS。

## 结果

一、一般情况

16例DEK-NUP214融合基因阳性AML患者发病时血常规（中位数）：WBC 12.5（0.8～99.0）×10^9^/L，5例患者WBC >20×10^9^/L，PLT 66（30～157）×10^9^/L，多为轻、中度贫血，中位骨髓原始细胞比例为0.568（0.225～0.830）。按照FAB分型，急性单核细胞白血病（M5）1例，急性粒-单核细胞白血病（M4）2例，急性粒细胞白血病部分分化型（M2）13例。染色体核型R显带分析，正常核型4例，异常核型12例，其中10例检出t（6;9）（p23;q34）。16例患者多重巢式PCR检测DEK-NUP214融合基因均为阳性。全部患者均行二代测序检测51种白血病高频基因突变，突变比例最高的基因是FLT3（68.8％，11例），其中FLT3-ITD 10例，FLT3-TKD 1例；5例未检出FLT3基因突变，其中4例均检出N-RAS基因突变。详见表1。

**表1:** 16例DEK-NUP214融合基因阳性急性髓系白血病（AML）患者一般资料及诊断时疾病状态

例号	性别	年龄（岁）	FAB分型	骨髓原始细胞（%）	染色体核型	融合基因检测
1	女	24	M2	33	46,XX,t(6:9)(p23:q34)[20]	DEK-NUP214、FLT3-ITD阳性
2	男	47	M4	29.5	46,XY[12]	DEK-NUP214阳性
3	男	58	M2	79	46,XY[20]	DEK-NUP214、FLT3-ITD阳性
4	女	36	M2	23	46,XX,del(9)(q12q34)[10]	DEK-NUP214、NRAS、KRAS阳性
5	男	30	M2	57.5	46,XY[20]	DEK-NUP214、FLT3-ITD阳性
6	女	48	M5	77.5	46,XX,t(6:9)(p23:q34)[18]	DEK-NUP214、FLT3-ITD阳性
7	女	50	M2	22.5	46,XX,t(6:9)(p23:q34)[6]	DEK-NUP214、FLT3-TKD、TP53阳性
8	男	46	M2	56	46,XX,t(6:9)(p23:q34)[16]	DEK-NUP214、FLT3-ITD阳性
9	男	30	M2	75	45,XY,t(6;9)(p23;q34),-17,der(18)t(17;18)(q11.2;q23)[20]	DEK-NUP214、FLT3-ITD阳性
10	男	26	M2	78	48,XY,+8,+13,15p-[10]	DEK-NUP214、IDH2、NRAS阳性
11	男	23	M4	36	46,XY[20]	DEK-NUP214、FLT3-ITD阳性
12	女	41	M2	63.5	46,XX,t(6:9)(p23:q34)[16]	DEK-NUP214、FLT3-ITD、IDH2阳性
13	女	25	M2	83	46,XX,t(6:9)(p23:q34)[15]	DEK-NUP214、FLT3-ITD阳性
14	男	41	M2	75	45X,-Y,t(5;12)(q31;p13),t(6;9)(p23;q34)[20]	DEK-NUP214、IDH1、NRAS阳性
15	女	30	M2	24.5	46,XX,t(6:9)(p23:q34)[20]	DEK-NUP214、FLT3-ITD、TET2阳性
16	男	42	M2	35	46,XY,t(6;9)(p23;q34)[8]/47,idem,+13[4]	DEK-NUP214、WT1、NRAS阳性

注 M5：急性单核细胞白血病；M4：急性粒-单核细胞白血病；M2：急性粒细胞白血病部分分化型

二、移植前结果

16例DEK-NUP214融合基因阳性的AML患者中，1个疗程获CR 7例，≥2个疗程获CR 8例，总缓解率为93.7％（15/16），获得CR的15例患者接受1～4个疗程巩固化疗后均行allo-HSCT（单倍体移植5例，全相合同胞供者移植8例，全相合无关供者移植3例），诊断至移植的中位时间为5（3～7）个月。

三、移植特征

移植前疾病状态：CR 15例（7例患者DEK-NUP214融合基因未转阴），NR 1例。全部患者均获得造血重建，中位粒细胞植活时间为11（10～15）d，中位血小板植活时间为13（10～22）d。发生巨细胞病毒血症3例（1例出现巨细胞病毒肺炎），EB病毒血症3例（无淋巴细胞增殖性疾病发生），经积极治疗后病毒均转阴。移植后6例（37.5％）患者发生急性GVHD，其中Ⅰ、Ⅱ、Ⅲ、Ⅳ度分别为1例（16.7％）、2例（33.3％）、1例（16.7％）、2例（33.3％）。移植100 d后9例（56.2％）发生慢性GVHD，其中局限型 6例、广泛型 3例。8例经积极治疗后症状好转，病情稳定，1例广泛型慢性GVHD患者发生细菌、真菌、病毒混合肺部感染并死于呼吸衰竭。详见表2。

**表2:** 16例DEK-NUP214融合基因阳性急性髓系白血病（AML）患者移植特征及随访结果

例号	供者类型	移植前疾病状态	移植前DEK-NUP214	干细胞来源	GVHD预防	CMV感染	EBV感染	aGVHD	cGVHD	复发	转归	DFS(月)	OS(月)
1	单倍体	CR	阴性	骨髓+外周血+脐血	CsA+MTX+MMF+rATG	阴性	阴性	皮肤（Ⅳ度）	局限型	否	存活	66.9	66.9
2	全相合同胞	CR	阴性	外周血	CsA+MTX	阴性	阴性	无	局限型	否	存活	63	63
3	单倍体	CR	阳性	骨髓+外周血+脐血	CsA+MTX+MMF+rATG	阴性	阳性	无	局限型	否	存活	59.3	59.3
4	全相合同胞	CR	阳性	外周血	CsA+MTX	阴性	阴性	皮肤、肠道（Ⅱ度）	广泛型	否	存活	39.1	39.1
5	全相合同胞	CR	阳性	外周血	CsA+MTX	阴性	阴性	无	无	否	存活	35.5	35.5
6	全相合同胞	CR	阳性	外周血	CsA+MTX	阴性	阴性	无	无	否	存活	24.7	24.7
7	单倍体	CR	阴性	外周血+脐血	CsA+MTX+MMF+rATG	阴性	阳性	无	无	否	存活	20.8	20.8
8	全相合同胞	NR	阳性	外周血	CsA+MTX	阴性	阴性	无	无	是	死亡	6.3	13.1
9	全相合同胞	CR	阴性	外周血	CsA+MTX	阳性	阴性	皮肤、肠道（Ⅲ度）	广泛型	否	存活	17.0	17.0
10	全相合无关	CR	阴性	外周血	CsA+MTX+MMF+rATG	阳性	阳性	肠道（Ⅱ度）	无	否	存活	42.3	42.3
11	单倍体	CR	阴性	骨髓+外周血+脐血	CsA+MTX+MMF+rATG	阳性	阴性	皮肤、肠道（Ⅳ度）	广泛型	否	死亡	5.9	5.9
12	单倍体	CR	阳性	骨髓+外周血+脐血	CsA+MTX+MMF+rATG	阴性	阴性	无	无	是	死亡	14.4	17.7
13	全相合同胞	CR	阴性	外周血	CsA+MTX	阴性	阴性	无	局限型	否	存活	36.6	36.7
14	全相合无关	CR	阳性	外周血	CsA+MTX+MMF+rATG	阴性	阴性	无	无	是	存活	3.5	3.7
15	全相合无关	CR	阴性	外周血	CsA+MTX+MMF+rATG	阴性	阴性	皮肤（Ⅰ度）	局限型	否	存活	3.2	3.2
16	全相合同胞	CR	阴性	外周血	CsA+MTX	阴性	阴性	无	局限型	否	存活	4.6	4.6

注 CR：完全缓解；NR：未缓解；MTX：甲氨蝶呤；CsA：环孢素A；rATG：兔抗人胸腺细胞球蛋白；CMV：巨细胞病毒；EBV：EB病毒；aGVHD：急性移植物抗宿主病；cGVHD：慢性移植物抗宿主病；DFS：无病生存；OS：总生存

四、生存分析

3例（18.8％）患者移植后出现疾病复发，其中微小残留病复发1例（移植后105 d）、血液学复发2例（移植后321 d、479 d）。2例血液学复发患者分别在形态学复发前48 d、132 d出现DEK-NUP214融合基因转阳。1例患者通过减停免疫抑制剂、阿扎胞苷治疗，但DEK-NUP214融合基因表达进行性升高，后出现形态学复发，再诱导化疗失败，死于本病。另外1例发现DEK-NUP214融合基因转阳后未予特殊干预，1月余后复查骨髓示血液学复发，放弃治疗后死亡。1例微小残留病复发患者通过减停免疫抑制剂，IDH1抑制剂（艾伏尼布）联合阿扎胞苷治疗中，目前骨髓暂未复查，密切随访中。3例患者死亡，2例死因为疾病复发，1例广泛型慢性GVHD发生重症肺部细菌、真菌、病毒混合感染，后死于呼吸衰竭，死亡时间分别为移植后5.9、13.1、17.7个月。中位随访时间为22.8（3.2～66.9）个月，预期3年OS率为（76.2±6.6）％，3年DFS率为（76.9±6.8）％。

## 讨论

t（6;9）（p23;q34）是血液系统肿瘤中少见的随机染色体异常，由Rowley和Poter在1976年在1例AML患者中发现[6]，后来1992年发现该染色体异位形成DEK-NUP214（DEK-CAN）融合蛋白。FAB分型中，DEK-NUP214融合蛋白与AML-M2和M4高度相关，我们这组病例中M2 13例（81.2％），M4 2例（12.5％），与既往文献报道一致。本组病例染色体R显带核型分析发现，正常核型4例，异常核型12例，其中10例患者检出t（6;9）（p23;q34）。染色体核型分析尽管能发现具体的易位染色体，但存在细胞增殖不良导致分析失败的可能，且不能检出隐匿性易位，但逆转录聚合酶链式反应（RT-PCR）法可检出DEK-NUP214融合基因。我们这组病例使用RT-PCR法均检出DEK-NUP214融合基因。因此，在临床工作中，联合应用这两种检测方法可以最大程度防止漏诊。

多项研究发现，DEK-NUP214融合基因阳性AML患者FLT3基因突变检出率高达70％以上，尤其是FLT3-ITD基因突变的检出率较其他AML患者高3倍[7]–[8]。本组病例中11例（66.7％）患者检出FLT3基因突变，其中10例为FLT3-ITD突变，与既往文献报道相似。我们还发现，5例未检出FLT3基因突变的患者中，4例检出N-RAS基因突变，可能提示N-RAS基因与FLT3基因的互排性，但需要更多的病例来验证。大量临床研究发现伴FLT3-ITD突变的AML患者预后差，FLT3抑制剂联合标准方案化疗可提高患者的缓解率[9]–[10]，为后续allo-HSCT提供机会，且多项研究证实移植后FLT3抑制剂维持治疗可明显减少复发及死亡风险[11]。本研究中存在FLT3基因突变的部分病例加用FLT3抑制剂（索拉非尼或富马酸吉瑞替尼）靶向治疗，总缓解率达93.7％，治疗效果满意。

研究DEK-NUP214蛋白功能时发现，该融合蛋白只是对个别造血干细胞存在诱发白血病作用，还具有维持白血病细胞干性的功能[12]。DEK-NUP214阳性AML患者化疗效果差、复发率高可能与此有关。Sandahi等[13]研究发现，DEK-NU214阳性儿童AML患者第一次CR（CR_1_）时进行allo-HSCT 5年无事件生存率、OS率均为68％，而单纯化疗组为18％、54％。Garçon等[14]对5例DEK-NUP214阳性AML患者行allo-HSCT，4例中位随访时间达到18.5个月，均无病生存，预后良好。国内高梦鸽等[15]对15例DEK-NUP214阳性AML的患者进行回顾性分析，allo-HSCT OS率、DFS率分别为60.5％、61.5％，与该中心标准危险分层AML患者的总体OS率大致相当。本研究大部分DEK-NUP214融合基因阳性AML患者在CR1状态下行allo-HSCT，移植后3年OS率为（76.2±6.6）％，3年DFS率为（76.9±6.8）％，取得了不错的效果。分析其原因，我们这组病例较早减停免疫抑制剂，后期56.2％的病例出现慢性GVHD，带来持续的移植物抗白血病反应。对于未发生持续慢性GVHD患者采用去甲基化药物（地西他滨或阿扎胞苷）或者FLT3抑制剂维持治疗，对控制复发及延长生存起一定作用。3例死亡患者中2例死于本病复发，是影响总体预后的主要因素。我们发现2例血液学复发患者分别在形态学复发前48、132 d出现DEK-NUP214融合基因转阳，常规治疗未能控制病情，这提示我们需要进一步探索治疗方法。

总之，DEK-NUP214阳性AML是一类少见的白血病类型，尽早进行allo-HSCT可能有助于改善预后。本研究病例数较少，以上结论尚需开展多中心研究加以验证。
